# Television screen time, but not computer use and reading time, is associated with cardio-metabolic biomarkers in a multiethnic Asian population: a cross-sectional study

**DOI:** 10.1186/1479-5868-10-70

**Published:** 2013-05-30

**Authors:** Ei Ei Khaing Nang, Agus Salim, Yi Wu, E Shyong Tai, Jeannette Lee, Rob M Van Dam

**Affiliations:** 1 Saw Swee Hock School of Public Health, National University of Singapore, Singapore, Republic of Singapore; 2 Department of Mathematics and Statistics, La Trobe University, Bundoora, Victoria, Australia; 3 Department of Medicine, National University Health System, Singapore, Republic of Singapore

**Keywords:** Physical activity, Sedentary behaviour, Television screen time, Cardio-metabolic biomarkers

## Abstract

**Background:**

Recent evidence shows that sedentary behaviour may be an independent risk factor for cardiovascular diseases, diabetes, cancers and all-cause mortality. However, results are not consistent and different types of sedentary behaviour might have different effects on health. Thus the aim of this study was to evaluate the association between television screen time, computer/reading time and cardio-metabolic biomarkers in a multiethnic urban Asian population. We also sought to understand the potential mediators of this association.

**Methods:**

The Singapore Prospective Study Program (2004–2007), was a cross-sectional population-based study in a multiethnic population in Singapore. We studied 3305 Singaporean adults of Chinese, Malay and Indian ethnicity who did not have pre-existing diseases and conditions that could affect their physical activity. Multiple linear regression analysis was used to assess the association of television screen time and computer/reading time with cardio-metabolic biomarkers [blood pressure, lipids, glucose, adiponectin, C reactive protein and homeostasis model assessment of insulin resistance (HOMA-IR)]. Path analysis was used to examine the role of mediators of the observed association.

**Results:**

Longer television screen time was significantly associated with higher systolic blood pressure, total cholesterol, triglycerides, C reactive protein, HOMA-IR, and lower adiponectin after adjustment for potential socio-demographic and lifestyle confounders. Dietary factors and body mass index, but not physical activity, were potential mediators that explained most of these associations between television screen time and cardio-metabolic biomarkers. The associations of television screen time with triglycerides and HOMA-IR were only partly explained by dietary factors and body mass index. No association was observed between computer/ reading time and worse levels of cardio-metabolic biomarkers.

**Conclusions:**

In this urban Asian population, television screen time was associated with worse levels of various cardio-metabolic risk factors. This may reflect detrimental effects of television screen time on dietary habits rather than replacement of physical activity.

## Background

The prevalence of diabetes mellitus is rising globally with an expected 552 million cases of diabetes worldwide by 2030 of which more than 60% are from Asia [1]. The benefits of regular physical activity for the prevention and treatment of type 2 diabetes are well established [2]. Recently, evidence has been emerging that sedentary behaviour has adverse effect on health independent of physical activity [3-5].

Television (TV) viewing time has been used as a measure of sedentary behaviour and several studies have shown an association between TV viewing time and cardiovascular risk factors, the metabolic syndrome, and type 2 diabetes [6-11].

However results have not been entirely consistent with other studies, that did not find an association between TV viewing time and cardiovascular risk factors [12,13].In addition, most previous studies that evaluated whether the association for TV viewing was independent of physical activity adjusted only for leisure time physical activity, but did not consider non-leisure physical activity[6-11]. Furthermore, it is possible that TV viewing, as an epidemiologic construct, comprises more than sedentary behaviour [14,15]. For example, food and beverage consumption during TV viewing and exposure to food commercials may lead to worse dietary habits. Few studies have evaluated whether dietary intakes may mediate effects of TV viewing on cardio-metabolic risk factors [10,11,16].

Hence, the aim of our study was to examine the association of TV screen time with cardio-metabolic biomarkers in an Asian population considering a wide range of potential confounders and mediators including total physical activity, dietary factors and body mass index (BMI). We also examined associations between computer/reading time and cardio-metabolic biomarkers to evaluate whether TV screen time may have unique effects.

## Methods

The Singapore Prospective Study Program, conducted between 2004 and 2007, was a population-based study in a multiethnic population (Chinese, Malay and Indian) in Singapore. The participants in this study took part in four previous population-based cross-sectional surveys carried out between 1982 and 1998 in Singapore. Detailed descriptions of these studies have been previously published [17]. Briefly, these previous surveys were all conducted in a random sample of individuals from the Singapore population, with disproportionate sampling stratified by ethnicity to increase the numbers for ethnic minority groups (Malays and Asian Indians). Participants who were deceased at time of follow-up (through data-linkage with the Registry of Births and Deaths) (n= 559), who had emigrated (n=6) or who had an error in their identity card number (n=102) could not be included in the follow-up. Three home visits on three different occasions including one weekend and weekday were made before a participant was deemed non-contactable. 2306 participants were regarded as non-contactable. Hence, 7774 participants remained from a total of 10747 participants from the four surveys. Of the remaining participants, thirty (0.3%) refused to participate in the follow-up assessments. Thus a total of 7744 participants, mean age of 49.7 years old (age ranged from 24.4 to 94.8 years old), participated in the study.

A questionnaire was administered by trained staff at the participant’s home. Questionnaires were in English and when needed interviewers provided additional explanation in Chinese, Malay or Tamil. All the interviewed participants were subsequently invited to attend a health examination for additional tests and collection of blood samples shortly after the home visit. A total of 7,744 (76.8% response rate) were interviewed, of which 5,163 (66.7% response rate, or 51.3% of total eligible participants) participants attended the health examination. Ethics approval was obtained from the Institutional Review Boards of National University of Singapore and Singapore General Hospital. Informed consent was obtained from all participants before conduct of study.

### Assessment of TV screen time, computer time and reading time

TV screen time was assessed by asking participants “Currently how many hours per day do you spend watching television or playing computer/handheld video games on the television screen?” Computer and reading time were assessed by the following questions; 1) “currently how many hours per day do you spend using the computer?” and 2) “Currently, how many hours per day do you read and write?”

### Assessment of risk factors

For health examination, participants were examined following a 10-hour overnight fast. Venous blood was drawn and collected in plain and fluoride oxalate tubes and stored at 4°C for a maximum of 4 hours prior to processing. All biochemical analyses on blood were carried out at the National University Hospital Referral Laboratory, which is accredited by the College of American Pathologists. Serum total cholesterol (TC), triglyceride (TG), and high density lipoprotein cholesterol (HDL-C) were measured using an automated autoanalyzer (ADVIA 2400, Bayer Diagnostics). Low density lipoprotein cholesterol (LDL-C) levels were calculated using the Friedewald formula. Fasting plasma glucose (FPG) was also assayed using enzymatic methods (ADVIA 2400, Bayer Diagnostics) using blood collected in fluoride oxalate tubes. High sensitivity-C-reactive protein (hs-CRP) was measured using immunoturbidimetric assay (Roche Integra 400). Insulin was assayed by microparticle enzyme immunoassay using the Bayer ADVIA Centaur chemiluminescent assay. Insulin resistance was assessed by homeostasis model assessment [Insulin resistance, HOMA-IR= (fasting insulin x fasting glucose)/22.5]. Fasting serum adiponectin were determined using a sandwich enzyme-linked immunosorbent assay using an antibody specific for all multimeric forms of human adiponectin (Daiichi Pure Chemicals, Japan).

The range of intra and inter-day coefficient of variation were TC (0.80-1.57% and 0.93-1.15%), TG (0–3.85% and 1.27-3.40%), HDL-C (0.56-0.65% and 1.18-2.00%), FPG (0–0.93% and 1.68-1.83%), hs-CRP (0.60–1.30 and 2.30–3.10%), insulin (2.40-4.00% and 3.85-6.29%), adiponectin (18.09% and 15.94%) and high molecular weight (HMW) adiponectin (6.79% and 18.35%) respectively.

Two readings of blood pressure were taken from participants after five minutes rest using an automated blood pressure monitor (Dinamap Pro100V2; Criticon, Norderstedt, Germany). A third reading was performed if difference between two readings of systolic blood pressure was greater than 10 mmHg or diastolic blood pressure was greater than 5 mmHg. Mean values of the closest two readings were calculated. The inter- and intra-observer coefficients of variation for systolic blood pressure were ranged from 0.51 to 10.20% and 0 to 2.50% whilst it was 0.41 to 7.50% and 0 to 2.50% for diastolic blood pressure.

### Assessment of covariates

Height was measured without shoes using a wall-mounted stadiometer. Weight was measured in light clothing using the same digital scale (SECA, model 782 2321009; Vogel& Halke, Germany) for all participants. Participants were instructed to remove any objects such as keys and mobile phones before measurement. BMI was obtained by dividing weight (kg) by the square of height (m).

Demographic, physical activity, dietary intake, medical history and other lifestyle factors were assessed by an interviewer-administered questionnaire. Dietary intake was assessed by a semi-quantitative 169-item validated food frequency questionnaire that is also used in the National Nutrition Surveys [18] and physical activity was assessed by a validated Singapore prospective study physical activity questionnaire which covered transportation, leisure time, occupation and household activities [19]. The detail method of assessing physical activity was described elsewhere [19,20]. Briefly, the participants reported the type, frequency and duration of various activities in the transportation, occupation, leisure time, and household domain. Transportation activities included walking and cycling and occupational activity included light, moderate and vigorous occupational activity. Leisure time activities included 48 specific activities, household activities included 15 specific activities and also asked about possible other activities for these two domains by open-ended questions. A metabolic equivalent of task (MET) value was assigned to each type of reported activity according to the compendium by Ainsworth et al. [21]. Then physical activity level per week for each activity was calculated as frequency per week x duration in hours per day x intensity (METs). For example, if a participant reported bowling activity for 60 minutes per day for 3 days a week, then the participant’s physical activity level for this bowling activity was calculated as 3 days x 1 hour x 3 METs, resulting in 9 MET-hours/week. Total physical activity was calculated as the sum of MET-hours/week spend on all activities. Light intensity was defined as 1.6 to 2.9 METs, moderate intensity was defined as 3.0 to 6.0 METs and vigorous intensity was defined as more than 6.0 METs [21,22]. Time spent on light, moderate, and vigorous activity was derived from the sum of time spent on all activities at that level from all domains.

### Statistical analysis

From the 5163 participants who attended the health examination, we excluded participants with known diabetes (N=813), known hypertension (N=1742), cardiovascular disease (N=399), self-reported angina (N=236) and cancer (N=75) to avoid potential reverse causation. In addition, we also excluded individuals who were pregnant (N=2), had invalid food frequency data (N=413), those who reported ethnicity other than Chinese, Malay or Indian (N=3) and missing other exposure variables (N=196). For analysis of lipids, glucose, hs-CRP, adiponectin and HOMA-IR, we excluded participants on lipid lowering medication (N=955). The participants might be excluded for one or more reasons. A total of 1835 participants were excluded for the outcome of systolic and diastolic blood pressure and 1956 participants were excluded for the other outcomes. Hence, there are 3305 participants, mean age of 47.1 years old (age ranged from 24.4 to 94.8 years old), included in the analysis for blood pressure and 3184 for the other outcomes (mean age of 46.7 years old, age ranged from 24.4 to 94.8 years old) .

HDL-c, FPG, TG, hs-CRP, HMW and total adiponectin, and HOMA-IR were log-transformed to achieve a normal distribution. The energy percentage of carbohydrate and protein were calculated as the calories from carbohydrate/ protein divided by total calories intake. For Table 1, we calculated means and standard deviations for continuous variables and proportions for categorical variables in each category of TV screen time. The P value for trend for the association between TV screen time and age was assessed by linear regression and the P values for trend between TV screen time and categorical variables was assessed by the Cochran-Mantel-Haenszel statistic. In Table 2, the Pearson’s partial correlation coefficients between potential mediators and TV screen time (truncated at 4 standard deviation i.e. TV screen time greater than the mean plus 4 times the SD was replaced with the value of the mean plus 4 times the 4SD. There were no values lower than the mean minus 4 times the SD) were calculated, adjusting for age, sex, ethnicity and education. In Table 3, multiple linear regression analysis was used to assess the association between category of TV screen time and risk factors with the lowest category as a reference group and the adjusted means for each category of TV screen time were presented in the first 4 columns. For the adjusted means of log transformed outcomes, we back-transformed (exponentiated) the adjusted means to obtain adjusted geometric means of the outcomes. To test the overall association of TV screen time with risk factors, we also ran multiple linear regression analysis with TV screen time (truncated at 4 standard deviations) as continuous exposure variable and cardio-metabolic biomarkers as outcome variables.

**Table 1:** Characteristics of the study population by TV screen time in 3305 Singaporeans

	**TV screen time (hours/day)**				
	**<1**	**1- 1.99**	**2-2.99**	**>=3**	**p for trend**
N (%)	488 (14.77)	1031 (31.20)	977 (29.56)	809 (24.48)	
Age, years (mean±SD)	47.06 ± 10.62	46.20 ± 10.44	46.67 ± 10.43	48.58 ± 11.48	<0.0001
Sex (N, %)					
Male	242 (49.59)	503 (48.79)	450 (46.06)	328 (40.54)	0.0002
Female	246 (50.41)	528 (51.21)	527 (53.94)	481 (59.46)	
Ethnicity (N, %)					
Chinese	356 (72.95)	719 (69.74)	666 (68.17)	546 (67.49)	0.22
Malay	60 (12.30)	175 (16.97)	184 (18.83)	150 (18.54)	
Indian	72 (14.75)	137 (13.29)	127 (13.00)	113 (13.97)	
Highest level of education (N, %)					
None/ primary	105 (21.56)	175 (16.97)	195 (19.96)	235 (29.05)	<0.0001
Secondary	170 (34.91)	402 (38.99)	390 (39.92)	341 (42.15)	
Vocational training	87 (17.86)	241 (23.38)	237 (24.26)	155 (19.16)	
University	125 (25.67)	213 (20.66)	155 (15.86)	78 (9.64)	
Current Employment status (N, %)					
Yes	389 (79.71)	848 (82.25)	761(77.89)	484 (59.83)	<0.0001
No	99 (20.29)	183 (17.75)	216 (22.11)	325 (40.17)	
Cigarette smoking (N, %)					
Never smoker	387 (79.30)	830 (80.50)	757 (77.48)	640 (79.11)	0.96
Current smoker	59 (12.09)	128 (12.42)	129 (13.20)	122 (15.08)	
Ex-smoker	42 (8.61)	73 (7.08)	91 (9.31)	47 (5.81)	
Current alcohol consumption (N, %)	81 (16.60)	173 (16.78)	190 (19.45)	139 (17.18)	0.49

**Table 2:** Pearson's partial correlation coefficient between TV screen time and lifestyle factors

	**TV screen time (hours/day)**
Body Mass Index (kg/m^2^)	0.085‡
Total calorie intake (kcal/d)	0.090‡
Cholesterol intake (mg per 1000 kcal)	0.070†
Fibre intake (g per 1000 kcal)	−0.082‡
Carbohydrate intake (energy %)	−0.092‡
Protein intake (energy %)	0.017
Polyunsaturated: saturated ratio of fat	−0.012
**Physical activity**	
Total physical activity (MET-hours/week)	−0.012
Light physical activity (MET-hours/week)	−0.003
Moderate physical activity (MET-hours/week)	−0.011
Vigorous physical activity (MET-hours/week)	−0.006
Partial correlation adjusted for age, ethnicity, sex and education	
†p value ≤ 0.01,‡p value ≤ 0.0001	
None of the correlations had a P value >0.01 and <= 0.05	

**Table 3:** Adjusted mean (and 95%CI) of cardio-metabolic biomarkers by TV screen time

	**TV screen time (hours/day)**					**p for trend**
	**<1 (Reference category)**		**1- 1.99**	**2-2.99**	**>=3**	
**Median of TV screen time**		**0.5**	**1**	**2**	**3**	
N (%)		488(14.77)	1031(31.20)	977(29.56)	809(24.48)	
Systolic blood pressure (mmHg)	Model 1	126.1	126.9	126.6	128.6	0.002
		(124.6,127.5)	(125.9,127.9)	(125.6,127.6)	(127.5,129.7)†	
	Model 2	126.2	127.0	126.6	128.3	0.01
		(124.8,127.6)	(126.1,128.0)	(125.6,127.6)	(127.2,129.4)*	
	Model 3	126.6	127.3	126.5	127.9	0.19
		(125.2,127.9)	(126.3,128.2)	(125.6,127.5)	(126.8,129)	
Diastolic blood pressure (mmHg)	Model 1	76.1 (75.3,76.9)	76.0 (75.5,76.6)	76.1 (75.5,76.6)	76.5 (75.9,77.2)	0.37
	Model 2	76.1 (75.3,77.0)	76 .0 (75.4,76.6)	76.0 (75.4,76.6)	76.6 (76.0,77.3)	0.21
	Model 3	76.3 (75.5,77.1)	76.2 (75.6,76.7)	75.9 (75.4,76.5)	76.4 (75.8,77.1)	0.86
HDL-c (mmol/L)	Model 1	1.42 (1.39,1.45)	1.42 (1.40,1.44)	1.41 (1.39,1.43)	1.39 (1.37,1.41)	0.004
	Model 2	1.42 (1.39,1.44)	1.41 (1.40,1.43)	1.41 (1.39,1.43)	1.39 (1.37,1.41)	0.01
	Model 3	1.41 (1.38,1.44)	1.41 (1.39,1.43)	1.41 (1.39,1.43)	1.40 (1.38,1.42)	0.14
LDL-c (mmol/L)	Model 1	3.18 (3.11, 3.25)	3.22 (3.17, 3.27)	3.27 (3.22, 3.32)	3.31 (3.25, 3.36)†	0.004
	Model 2	3.19 (3.12, 3.26)	3.22 (3.17, 3.27)	3.26 (3.21, 3.31)	3.30 (3.24, 3.36)*	0.01
	Model 3	3.20 (3.13, 3.27)	3.23 (3.19, 3.28)	3.26 (3.21, 3.31)	3.29 (3.23, 3.34)	0.08
Cholesterol (mmol/L)	Model 1	5.19 (5.11, 5.27)	5.23 (5.18, 5.29)	5.28 (5.23, 5.34)	5.34 (5.28, 5.40)†	0.002
	Model 2	5.19 (5.11, 5.27)	5.24 (5.18, 5.29)	5.28 (5.23, 5.34)	5.33 (5.27, 5.40)†	0.008
	Model 3	5.20 (5.12, 5.28)	5.25 (5.19, 5.30)	5.28 (5.22, 5.33)	5.32 (5.25, 5.38)*	0.06
Fasting plasma glucose (mmol/L)	Model 1	4.76 (4.69, 4.82)	4.76 (4.72, 4.81)	4.80 (4.75, 4.85)	4.80 (4.75, 4.85)	0.10
	Model 2	4.76 (4.69, 4.82)	4.76 (4.72, 4.81)	4.80 (4.75, 4.85)	4.80 (4.75, 4.85)	0.08
	Model 3	4.77 (4.71, 4.83)	4.77 (4.73, 4.82)	4.80 (4.75, 4.84)	4.78 (4.73, 4.83)	0.43
Triglycerides (mmol/L)	Model 1	1.05 (1.00, 1.09)	1.07 (1.04, 1.10)	1.09 (1.06, 1.13)	1.15 (1.11, 1.19)†	<0.0001
	Model 2	1.05 (1.01, 1.10)	1.08 (1.04, 1.11)	1.09 (1.06, 1.13)	1.14 (1.11, 1.18)†	<0.0001
	Model 3	1.07 (1.02, 1.11)	1.09 (1.06, 1.12)	1.09 (1.05, 1.12)	1.12 (1.09, 1.16)	0.04
hsCRP(mg/L)	Model 1	0.94 (0.85, 1.04)	1.03 (0.96, 1.10)	1.04 (0.96, 1.11)	1.20 (1.11, 1.30)‡	<0.0001
	Model 2	0.94 (0.85, 1.04)	1.03 (0.96, 1.11)	1.03 (0.96, 1.11)	1.20 (1.11, 1.30)‡	<0.0001
	Model 3	0.99 (0.90, 1.08)	1.07 (1.00, 1.14)	1.02 (0.95, 1.09)	1.13 (1.05, 1.21)*	0.10
High-molecular weight adiponectin (μg/mL)	Model 1	1.18 (1.10, 1.26)	1.13 (1.08, 1.18)	1.10 (1.05, 1.15)	1.08 (1.02, 1.14)*	0.048
	Model 2	1.17 (1.10, 1.25)	1.12 (1.07, 1.18)	1.10 (1.05,1.16)	1.09 (1.03, 1.15)	0.10
	Model 3	1.15 (1.07, 1.22)	1.10 (1.06, 1.16)	1.11 (1.06, 1.16)	1.12 (1.06, 1.18)	0.89
Total adiponectin (μg/mL)	Model 1	3.58 (3.42, 3.75)	3.38 (3.27, 3.48)*	3.32 (3.21,3.42)†	3.30 (3.18, 3.42)†	0.01
	Model 2	3.57 (3.41, 3.73)	3.37(3.27, 3.48)*	3.32 (3.22, 3.43)*	3.31 (3.19, 3.43)†	0.03
	Model 3	3.51 (3.36, 3.66)	3.33 (3.23, 3.43)*	3.34 (3.24, 3.44)	3.37 (3.26, 3.49)	0.52
HOMA-IR	Model 1	1.17 (1.10, 1.24)	1.23 (1.18, 1.28)	1.26 (1.21, 1.31)*	1.38 (1.32, 1.44)‡	<0.0001
	Model 2	1.17 (1.11, 1.24)	1.23 (1.18, 1.28)	1.26 (1.21, 1.31)*	1.37 (1.31, 1.44)‡	<0.0001
	Model 3	1.21 (1.15, 1.27)	1.26 (1.22, 1.31)	1.25 (1.20, 1.29)	1.32 (1.27, 1.38)†	0.047

Model 1: Adjusted for age, sex, ethnicity, education.

Model 2: Model 1 further adjusted for reading time, computer time, employment status, smoking, alcohol, parental history of diabetes, parental history of hypertension.

Model 3: Model 2 further adjusted for potential mediators including total physical activity, BMI, ratio of polyunsaturated-to-saturated fat intake, and intake of total energy, fibre, cholesterol, carbohydrate and protein.

HDL-c, fasting plasma glucose, triglycerides, hsCRP, high-molecular weight adiponectin, total adiponectin,and HOMA-IR were log transformed and the adjusted means were back transformed.

* p value ≤ 0.05, †p value ≤ 0.01,‡p value ≤ 0.0001.

p for trend: p value of linear regression for association of TV screen time (as a continuous variable) and outcome variables.

Model 1 was adjusted for age (years), sex, ethnicity (Chinese, Malay, Indian) and highest level of education (None/primary, secondary, vocational training, university). Model 2 was further adjusted for reading time (hours/week), computer time (hours/week), employment status (currently employed or not), cigarette smoking (never smoker, current smoker and ex-smoker), alcohol consumption (consumed alcohol in the past 3 months, yes/no), and parental history of diabetes and hypertension (yes/no). Additional adjustment for BMI (kg/m^2^), total physical activity level (MET-hours per week) and dietary factors including the ratio of polyunsaturated to saturated fat intake, total calorie intake (kcal/day), cholesterol intake (mg/1000 kcal), fibre intake (g/1000 kcal), and energy percentage of carbohydrate and protein was done in model 3 to understand the possible role of these factors in mediating the relationship between TV screen time and cardio-metabolic biomarkers. Similar analysis as presented for Table 2 and Table 3 for TV screen time were also done to assess the association of computer/reading time with cardio-metabolic biomarkers (Additional file 1 and Additional file 2).

**Figure 1:**
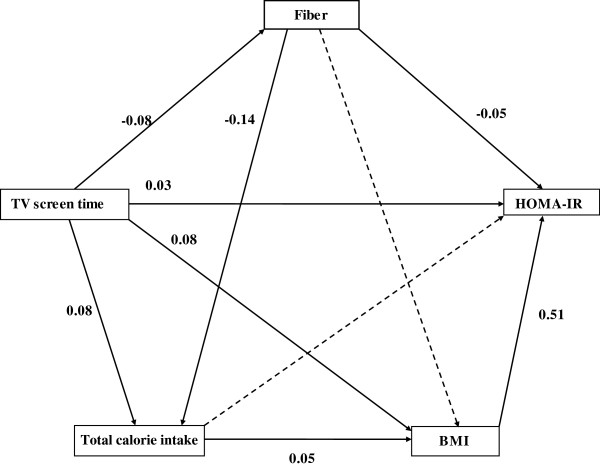
**Contribution of potential mediators to the association of TV screen time and HOMA-IR.** Standardized path coefficients are labelled on each path. The dotted arrows represent non-significant paths. Covariates including age, sex, ethnicity, education and total physical activity are not shown.

For Figure 1, path analysis was used to further examine the role of mediators in the association between TV screen time and HOMA-IR. Path analysis is an extension of multiple regression analysis that can simultaneously assess the strength and direction of the interrelationships among exposures, potential mediators and outcomes [21]. The path model was constructed based on previous research findings for the directions of the studied effects. It was modified by removing non-significant paths (p values > 0.05) and path analysis was performed again based on the reduced model. Results of the path analysis are presented as standardized path coefficients, indicating the difference in outcome variables (in standard deviation units) for a one standard deviation increment in exposure variables. The fit of the model was evaluated by fit statistics; normed fit index (NFI), comparative fit index (CFI) and root mean square error of approximation (RMSEA). Indirect effect was calculated by multiplying the coefficients of the paths involved. IBM SPSS Amos 19 (SPSS Inc, Chicago, Illinois) was used to conduct path analysis and STATA 11 (STATA Corp, College station, Texas) was used to run all the other analyses.

**Figure 2:**
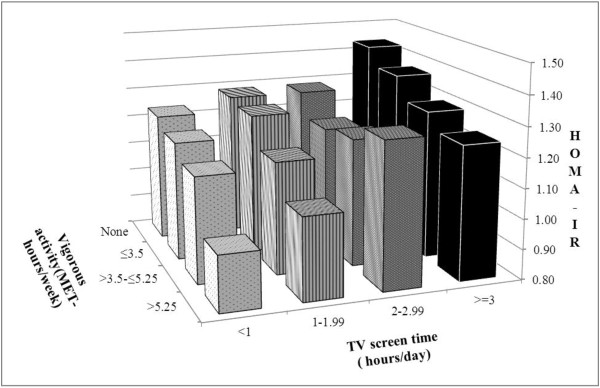
**Adjusted mean of the HOMA -IR by categories of TV screen time and vigorous activity.** Estimates were adjusted for age, sex, ethnicity, education, reading time, computer time, employment status, cigarette smoking, alcohol use, and parental history of diabetes and hypertension. As compared with the category with the least TV screen time (<1 hour/day) and the largest amount of vigorous activity (>5.25 MET-hours/week) all categories had significantly higher HOMA-IR values (P<0.05) except the category of having TV screen time <1 hour/day and vigorous activity ≤3.5 MET-hours/week, the category of having TV screen time <1 hour/day and vigorous activity >3.5-≤5.25 MET-hours/week, and the category of having TV screen time 1–1.99 hours/day and vigorous activity>5.25 MET-hours/week.

For Figure 2, 16 categories of TV screen time-vigorous activity were derived reflecting all combinations of 4 categories of TV screen time and 4 categories of vigorous activity. Multiple linear regressions was performed to assess the association between combinations of TV screen time and vigorous activity in relation to HOMA-IR with having little TV screen time (<1hour/day) and high vigorous activity (>5.25 MET-hours/week) as the reference category.

Interaction between TV screen time and vigorous activity was tested by including a multiplicative interaction term in a multivariable regression model (adjusting for the same confounders as for Figure 2).

## Results

Table 1 shows characteristics of the participants according to TV screen time. Participants who were older, female, had a lower education level, or were unemployed were more likely to have longer TV screen time. Ethnicity, cigarette smoking and alcohol drinking were not significantly associated with TV screen time.

Table 2 shows partial correlations between TV screen time and potential mediators of its adverse health effects, adjusted for age, sex, ethnicity and education. TV screen time was associated with higher BMI, total calorie intake, and cholesterol intake and with lower fibre and carbohydrate intake. No significant correlations were found between TV screen time and protein intake, the polyunsaturated-to-saturated fat ratio, and total, light, moderate and vigorous physical activity.

Table 3 shows the association between TV screen time and cardio-metabolic biomarkers. There were significant associations between TV screen time and several biomarkers. In the model that was only adjusted for socio-demographic variables (model 1), TV screen time was associated with significantly higher systolic blood pressure (SBP), LDL-cholesterol, total cholesterol, triglycerides, hs-CRP, HOMA-IR, and lower total and high-molecular weight adiponectin levels. These associations did not substantially change after additional adjustment for other sedentary behaviours and potential confounders (which included reading time, computer time, employment status, smoking, alcohol, parental history of diabetes, parental history of hypertension), except the association with high-molecular weight adiponectin levels which became insignificant (model 2).

We next considered variables that could be mediators of the association between TV screen time and cardio-metabolic biomarkers. These were body mass index, total physical activity, total calorie intake, fibre intake, cholesterol intake, polyunsaturated to saturated fat ratio of diet, percentage of energy from carbohydrate and protein. When these variables were included in model 3, associations were attenuated, but remained statistically significant for triglycerides and HOMA-IR.

Similar analyses were carried out for other sedentary activities, namely time spent on reading or working on a computer (see Additional files 1 and 2). Additional file 1 shows the Pearson’s partial correlation between computer/reading time and potential mediators, adjusted for age, sex, ethnicity and education. Computer/reading time was associated with lower total, light and moderate physical activity. Although participants generally spent much more time on reading or using a computer than TV, none of the statistically significant associations with cardio-metabolic biomarkers that we observed for TV screen time were observed for computer/reading time (Additional file 2). In contrast, more computer/reading time was associated with lower diastolic blood pressure after adjustment for potential confounders and mediators (model 3).

Because BMI, diet, and physical activity appeared to be important mediators of the association between TV screen time and cardio-metabolic risk factors, we carried out path analysis to better clarify the roles of specific mediators that may be involved. This is shown in Figure 1 for the association between TV screen time and HOMA-IR. The fit statistics suggested that the model had a good fit (NFI: 0.99, CFI: 0.99, RMSEA: 0.05). TV screen time may have a direct (independent) effect on HOMA-IR as well as indirect effects acting through BMI and fibre intake. Of the association between TV screen time and HOMA-IR, 41.3% was direct, 52.0% was accounted for by BMI and 5.3% was accounted for by lower fibre intake. Other mediators including physical activity (total physical activity or light, moderate and vigorous activity separately) did not substantially contribute to the association between TV screen time and HOMA-IR. Adjustment for physical activity also did not appreciably change the association between TV screen time and other cardio-metabolic biomarkers (data not shown).

Figure 2 shows the joint effect of TV screen time and vigorous activity in relation to HOMA-IR after multivariable adjustment for the same potential confounders as in model 2 in Table 3. Participants with no vigorous activity and who had TV screen time for 3 hours or more per day had the highest mean HOMA-IR score (1.43; 95% CI 1.35-1.53), whereas those with the highest vigorous activity (>5.25 MET-hours/week) and the least TV screen time (<1hr/day) had the lowest adjusted HOMA-IR (0.98; 95% CI 0.85-1.14).

Associations for TV screen time and vigorous activity appeared to be additive without statistically significant interaction (p value=0.33).

## Conclusions

In this study in an urban Asian population, we found that TV screen time was associated with several biomarkers associated with an increased risk of cardiovascular and metabolic disease. Longer TV screen time was significantly associated with higher systolic blood pressure, LDL-cholesterol, total cholesterol, triglycerides, hs-CRP, HOMA-IR and lower HDL and adiponectin even after adjustment for potential confounders. This is consistent with the results of several studies conducted in western populations. In the AusDiab study of Australian adults, TV viewing time was associated with higher diastolic blood pressure, triglycerides and fasting insulin in women and fasting plasma glucose, 2-hr glucose and fasting insulin in men [22]. In the EPIC-Norfolk study of English adults aged 45–74 years, TV viewing time was associated with blood pressure, cholesterol, LDL, HDL and triglycerides [23].

Path analysis suggests that a large part of the association between TV screen time and cardio-metabolic biomarkers in our study was mediated through BMI, explaining more than half of the association between TV screen time and HOMA-IR. The findings related to obesity are consistent with data from the Health Survey for England, where it was reported that 28.6% to 60.3% of the association between TV time and cardiometabolic risk factors (systolic and diastolic blood pressure, HDL- cholesterol, total cholesterol) was explained by BMI [24]. These may relate to reduced energy expenditure due to increased TV viewing time. A randomized controlled trial in adults showed that restricting TV time for 3 weeks resulted in increased objectively measured energy expenditure [25]. Altered dietary intake may also contribute to the link between TV viewing and obesity. In our study, TV screen time was associated with a higher intake of calories, cholesterol, and a lower intake of fibre. In contrast, computer/reading time was not associated with dietary intakes. Studies have shown that TV viewing time is associated with unhealthy eating behaviours and obesity [11,26]. In addition to being associated to greater adiposity, dietary intakes associated with TV viewing also appeared to act through pathways that are independent of adiposity. In particular, our results suggested that lower fibre intake was a mediator of the association between TV screen time and HOMA-IR. This is consistent with previously reported associations between higher fibre intakes and lower insulin resistance [27,28]. However, based on the data from a cross-sectional study in isolation we cannot distinguish with certainty between mediators and confounders. We based our *a priori* model assumptions on results from previous studies [11,26].

Even after taking potential confounders and mediators into account, statistically significant associations remained for triglycerides and HOMA-IR. In our study, time spent on TV screen was not substantially associated with the amount of physical activity and TV screen time independently contributed to higher HOMA-IR (Figure 2). Healy et al. [29] also reported that detrimental effects of TV viewing time on metabolic risk factors (waist circumference, systolic blood pressure, 2-h plasma glucose fasting plasma glucose, triglycerides, and HDL-C) was observed even among the participants who met the physical activity guideline [29] and have suggested that sedentary activity is not just a marker for reduced physical activity, but may have some direct effect on health and should be considered separately from physical activity. In our study, forms of sedentary activity other than TV screen time (computer or reading time) were not associated with worse levels for biomarkers of cardiovascular and metabolic risk. The same finding was reported by the study done in Dutch young adults which found that TV time but not computer time was associated with cardiometabolic biomarkers [30]. It is possible that TV use differs from other sedentary activities. Energy expenditure during TV viewing might be lower than during computer use or reading [31]. Furthermore, TV viewing time is associated with other behavioural risk factors such as dietary intakes [32,33]. In the study done in Australian adults, it was found that TV viewing more than 3 hours per day was associated with abdominal obesity and the association was partly explained by the food and beverages intake during TV viewing time [34].

Our study has several strengths. This was the first large population-based study in Asian population that studied the association of TV screen time with metabolic traits. It was done in a multiethnic population, comprised of Chinese, Malays and Asian Indians. We had detailed information on potential mediators and confounders as well as relevant cardiometabolic risk factors. Limitations of our study included that we did not have data on eating habits during TV screen time though we considered overall dietary intakes. We also did not record sleep duration in our study. TV viewing time has been shown to be associated with short sleep duration which is in turn associated with cardio-metabolic biomarkers [35-37]. We did not capture TV screen time separately for weekday and weekend which might be different for working population. Furthermore, TV screen time, physical activity, diet and computer/reading time were self-reported and are thus affected by measurement error. Measurement error in TV screen time may have weakened the observed associations, but measurement errors in potential confounders may have led to residual confounding. As this is cross-sectional study, we cannot definitively infer causality. Even though we excluded participants with relevant diagnosed diseases to avoid reverse causation, we cannot exclude the possibility that being overweight led participants to spend more time on the TV screen.

In summary, our study confirms the association between TV screen time and cardio-metabolic biomarkers in a multiethnic Asian population. However, TV screen time is a complex construct that appears to include obesity and altered dietary intakes. The lack of association between computer/reading time and metabolic risk factors in study also suggests that the association between TV screen time and health reflects other lifestyle factors rather than sedentary time per se. Even after controlling for potential mediators, part of the association between TV screen time and triglyceride levels and insulin resistance remained unexplained in our study. Given the prominent role that TV has in modern society, further research is warranted to better understand why this behaviour is associated with cardio-metabolic health. This may facilitate the development of public health interventions that more effectively address the adverse consequences of TV screen time.

## Abbreviations

(TV): Television; (BMI): Body mass index; (TC): Total cholesterol; (TG): Triglyceride; (HDL-C): High density lipoprotein cholesterol; (LDL-C): Low density lipoprotein cholesterol; (FPG): Fasting plasma glucose; (hs-CRP): High sensitivity-C-reactive protein; (HOMA-IR): Homeostasis model assessment of insulin resistance; (HMW): High molecular weight; (NFI): Normed fit index; (CFI): Comparative fit index; (RMSEA): Root mean square error of approximation; (SBP): Systolic blood pressure.

## Competing interests

The authors declare that they have no potential competing interests.

## Authors’ contributions

EEKN analysed the data and wrote the manuscript. AS helped in data analysis and reviewed the manuscript. YW involved in data collection and reviewed the manuscript. EST and JL obtained the funding, designed and supervised the study and edited and reviewed the manuscript. RMVD helped in interpretation of the results, led the writing, edited and reviewed the manuscript. All authors read and approved the final manuscript.

## Supplementary Material

Additional file 1 Pearson’s partial correlation coefficient between computer/reading time and lifestyle factors. Click here for file

Additonal file 2 Adjusted mean (and 95%CI) of cardiometabolic biomarkers by computer/ reading time. Click here for file

## References

[B1] International Diabetes Federation International Diabetes Federation.Diabetes Atlas 2011 5 Brussels, Belgium: International Diabetes Federation

[B2] Pate R Pratt M Blair S Haskell W Macera C Bouchard C Physical activity and public health. A recommendation from the Centers for Disease Control and Prevention and the American College of Sports Medicine JAMA 1995 273 5 402 407 10.1001/jama.1995.03520290054029 7823386

[B3] Grontved A Hu FB Television viewing and risk of type 2 diabetes, cardiovascular disease, and all-cause mortality: a meta-analysis JAMA 2011 305 23 2448 2455 10.1001/jama.2011.812 21673296 PMC4324728

[B4] Hu FB Sedentary lifestyle and risk of obesity and type 2 diabetes Lipids 2003 38 2 103 108 10.1007/s11745-003-1038-4 12733740

[B5] Owen N Leslie E Salmon J Fotheringham MJ Environmental determinants of physical activity and sedentary behavior Exerc Sport Sci Rev 2000 28 4 153 158 11064848

[B6] Dunstan DW Salmon J Healy GN Shaw JE Jolley D Zimmet PZ Owen N Association of television viewing with fasting and 2-h postchallenge plasma glucose levels in adults without diagnosed diabetes Diabetes Care 2007 30 3 516 522 10.2337/dc06-1996 17327314

[B7] Dunstan DW Salmon J Owen N Armstrong T Zimmet PZ Welborn TA Cameron AJ Dwyer T Jolley D Shaw JE Physical activity and television viewing in relation to risk of undiagnosed abnormal glucose metabolism in adults Diabetes Care 2004 27 11 2603 2609 10.2337/diacare.27.11.2603 15504993

[B8] Dunstan DW Salmon J Owen N Armstrong T Zimmet PZ Welborn TA Cameron AJ Dwyer T Jolley D Shaw JE Associations of TV viewing and physical activity with the metabolic syndrome in Australian adults Diabetologia 2005 48 11 2254 2261 10.1007/s00125-005-1963-4 16211373

[B9] Fung TT Hu FB Yu J Chu NF Spiegelman D Tofler GH Willett WC Rimm EB Leisure-time physical activity, television watching, and plasma biomarkers of obesity and cardiovascular disease risk Am J Epidemiol 2000 152 12 1171 1178 10.1093/aje/152.12.1171 11130623

[B10] Hu FB Leitzmann MF Stampfer MJ Colditz GA Willett WC Rimm EB Physical activity and television watching in relation to risk for type 2 diabetes mellitus in men Arch Intern Med 2001 161 12 1542 1548 10.1001/archinte.161.12.1542 11427103

[B11] Hu FB Li TY Colditz GA Willett WC Manson JE Television watching and other sedentary behaviors in relation to risk of obesity and type 2 diabetes mellitus in women JAMA 2003 289 14 1785 1791 10.1001/jama.289.14.1785 12684356

[B12] Sidney S Sternfeld B Haskell WL Jacobs DR Jr Chesney MA Hulley SB Television viewing and cardiovascular risk factors in young adults: the CARDIA study Ann Epidemiol 1996 6 2 154 159 10.1016/1047-2797(95)00135-2 8775596

[B13] Ekelund U Brage S Griffin SJ Wareham NJ Objectively measured moderate- and vigorous-intensity physical activity but not sedentary time predicts insulin resistance in high-risk individuals Diabetes Care 2009 32 6 1081 1086 10.2337/dc08-1895 19252168 PMC2681043

[B14] Lowry R Wechsler H Galuska DA Fulton JE Kann L Television viewing and its associations with overweight, sedentary lifestyle, and insufficient consumption of fruits and vegetables among US high school students: differences by race, ethnicity, and gender J Sch Health 2002 72 10 413 421 10.1111/j.1746-1561.2002.tb03551.x 12617028

[B15] Pearson N Ball K Crawford D Mediators of longitudinal associations between television viewing and eating behaviours in adolescents Int J Behav Nutr Phys Act 2011 8 23 10.1186/1479-5868-8-23 21450065 PMC3078829

[B16] Wijndaele K Duvigneaud N Matton L Duquet W Delecluse C Thomis M Beunen G Lefevre J Philippaerts RM Sedentary behaviour, physical activity and a continuous metabolic syndrome risk score in adults Eur J Clin Nutr 2007 63 3 421 429 17971826 10.1038/sj.ejcn.1602944

[B17] Nang EE Khoo CM Tai ES Lim SC Tavintharan S Wong TY Heng D Lee J Is there a clear threshold for fasting plasma glucose that differentiates between those with and without neuropathy and chronic kidney disease?: the Singapore Prospective Study Program Am J Epidemiol 2009 169 12 1454 1462 10.1093/aje/kwp076 19406920

[B18] Cutter J Tan BY Chew SK Levels of cardiovascular disease risk factors in Singapore following a national intervention programme Bull World Health Organ 2001 79 10 908 915 11693972 PMC2566668

[B19] Nang EE Gitau Ngunjiri SA Wu Y Salim A Tai ES Lee J Van Dam RM Validity of the International Physical Activity Questionnaire and the Singapore Prospective Study Program physical activity questionnaire in a multiethnic urban Asian population BMC Med Res Methodol 2011 11 141 10.1186/1471-2288-11-141 21995825 PMC3212806

[B20] Khaing Nang EE Khoo EY Salim A Tai ES Lee J Van Dam RM Patterns of physical activity in different domains and implications for intervention in a multi-ethnic Asian population: a cross-sectional study BMC Public Health 2010 10 644 10.1186/1471-2458-10-644 20973981 PMC2976750

[B21] Gamborg M Andersen PK Baker JL Budtz-Jorgensen E Jorgensen T Jensen G Sorensen TI Life course path analysis of birth weight, childhood growth, and adult systolic blood pressure Am J Epidemiol 2009 169 10 1167 1178 10.1093/aje/kwp047 19357327 PMC2732973

[B22] Thorp AA Healy GN Owen N Salmon J Ball K Shaw JE Zimmet PZ Dunstan DW Deleterious associations of sitting time and television viewing time with cardiometabolic risk biomarkers: Australian Diabetes, Obesity and Lifestyle (AusDiab) study 2004–2005 Diabetes Care 2010 33 2 327 334 10.2337/dc09-0493 19918003 PMC2809275

[B23] Jakes RW Day NE Khaw KT Luben R Oakes S Welch A Bingham S Wareham NJ Television viewing and low participation in vigorous recreation are independently associated with obesity and markers of cardiovascular disease risk: EPIC-Norfolk population-based study Eur J Clin Nutr 2003 57 9 1089 1096 10.1038/sj.ejcn.1601648 12947427

[B24] Stamatakis E Hamer M The extent to which adiposity markers explain the association between sedentary behavior and cardiometabolic risk factors Obesity (Silver Spring) 2012 20 1 229 232 10.1038/oby.2011.209 21779091

[B25] Otten JJ Jones KE Littenberg B Harvey-Berino J Effects of television viewing reduction on energy intake and expenditure in overweight and obese adults: a randomized controlled trial Arch Intern Med 2009 169 22 2109 2115 10.1001/archinternmed.2009.430 20008695

[B26] Pearson N Biddle SJ Sedentary behavior and dietary intake in children, adolescents, and adults. A systematic review Am J Prev Med 2011 41 2 178 188 10.1016/j.amepre.2011.05.002 21767726

[B27] McKeown NM Meigs JB Liu S Saltzman E Wilson PW Jacques PF Carbohydrate nutrition, insulin resistance, and the prevalence of the metabolic syndrome in the Framingham Offspring Cohort Diabetes Care 2004 27 2 538 546 10.2337/diacare.27.2.538 14747241

[B28] Lau C Faerch K Glumer C Tetens I Pedersen O Carstensen B Jorgensen T Borch-Johnsen K Dietary glycemic index, glycemic load, fiber, simple sugars, and insulin resistance: the Inter99 study Diabetes Care 2005 28 6 1397 1403 10.2337/diacare.28.6.1397 15920058

[B29] Healy GN Dunstan DW Salmon J Shaw JE Zimmet PZ Owen N Television time and continuous metabolic risk in physically active adults Med Sci Sports Exerc 2008 40 4 639 645 10.1249/MSS.0b013e3181607421 18317383

[B30] Altenburg TM de Kroon ML Renders CM Hirasing R Chinapaw MJ TV time but not computer time is associated with cardiometabolic risk in Dutch young adults PLoS One 2013 8 2 e57749 10.1371/journal.pone.0057749 23460900 PMC3584035

[B31] Ainsworth BE Haskell WL Whitt MC Irwin ML Swartz AM Strath SJ O'Brien WL Bassett DR Jr Schmitz KH Emplaincourt PO Compendium of physical activities: an update of activity codes and MET intensities Med Sci Sports Exerc 2000 32 9 Suppl S498 504 10993420 10.1097/00005768-200009001-00009

[B32] Scully M Dixon H Wakefield M Association between commercial television exposure and fast-food consumption among adults Public Health Nutr 2009 12 1 105 110 10.1017/S1368980008002012 18339226

[B33] Bowman SA Television-viewing characteristics of adults: correlations to eating practices and overweight and health status Prev Chronic Dis 2006 3 2 A38 16539779 PMC1563980

[B34] Cleland VJ Schmidt MD Dwyer T Venn AJ Television viewing and abdominal obesity in young adults: is the association mediated by food and beverage consumption during viewing time or reduced leisure-time physical activity? Am J Clin Nutr 2008 87 5 1148 1155 18469233 10.1093/ajcn/87.5.1148

[B35] Bjorvatn B Sagen IM Oyane N Waage S Fetveit A Pallesen S Ursin R The association between sleep duration, body mass index and metabolic measures in the Hordaland Health Study J Sleep Res 2007 16 1 66 76 10.1111/j.1365-2869.2007.00569.x 17309765

[B36] Martinez-Gomez D Eisenmann JC Gomez-Martinez S Hill EE Zapatera B Veiga OL Marcos A Sleep duration and emerging cardiometabolic risk markers in adolescents AFINOS Study Sleep med 2011 12 10 997 1002 10.1016/j.sleep.2011.05.009 22036601

[B37] Wells JC Hallal PC Reichert FF Menezes AM Araujo CL Victora CG Sleep patterns and television viewing in relation to obesity and blood pressure: evidence from an adolescent Brazilian birth cohort Int J Obes (Lond) 2008 32 7 1042 1049 10.1038/ijo.2008.37 18347603

