# Supplementary material for: Television screen time, but not computer use and reading time, is associated with cardio-metabolic biomarkers in a multiethnic Asian population: a cross-sectional study
Source: Int J Behav Nutr Phys Act. 2013 May 30;10:70. doi: 10.1186/1479-5868-10-70 (PMC3680020; doi:10.1186/1479-5868-10-70)
Supplement: Additional file 1 — Pearson’s partial correlation coefficient between computer/reading time and lifestyle factors. [file 1479-5868-10-70-S1.doc]

| **Additional file 1. Pearson's partial correlation coefficient between computer/reading time and lifestyle factors** | |
| --- | --- |
|  | **Computer/reading time (hours/day)** |
| Body Mass Index (kg/m²) | -0.007 |
| Total calorie intake (kcal/d) | 0.004 |
| Cholesterol intake ( mg per 1000 kcal) | -0.017 |
| Fibre intake ( g per 1000 kcal) | 0.024 |
| Carbohydrate intake (energy %) | -0.028 |
| Protein intake (energy %) | -0.019 |
| Polyunsaturated: saturated ratio of fat | -0.019 |
|  |  |
| **Physical activity** |  |
| Total physical activity (MET-hours/week) | -0.158* |
| Light physical activity (MET-hours/week) | -0.120* |
| Moderate physical activity (MET-hours/week) | -0.108* |
| Vigorous physical activity (MET-hours/week) | -0.009 |
| Partial correlation adjusted for age, ethnicity , sex and education | |
| * p value ≤ 0.0001  None of the correlations had a P value >0.0001 and </= 0.05 |  |
