# Supplementary material for: Television screen time, but not computer use and reading time, is associated with cardio-metabolic biomarkers in a multiethnic Asian population: a cross-sectional study
Source: Int J Behav Nutr Phys Act. 2013 May 30;10:70. doi: 10.1186/1479-5868-10-70 (PMC3680020; doi:10.1186/1479-5868-10-70)
Supplement: Additonal file 2 — Adjusted mean (and 95%CI) of cardiometabolic biomarkers by computer/ reading time. [file 1479-5868-10-70-S2.doc]

**Additional file 2. Adjusted mean (and 95%CI) of cardio-metabolic biomarkers by computer/** reading time

|  |  | |  | **Computer/reading time(hours/day)** | | | | | | |  |  |  |
| --- | --- | --- | --- | --- | --- | --- | --- | --- | --- | --- | --- | --- | --- |
|  |  | |  | **1st quartile** | **2nd quartile** | **3rd quartile** | **4th quartile** | | **¶ p for trend** | |  |  |  |
|  |  | |  | **≤0.5** | **>0.5- ≤2** | **>2-≤7** | **>7** | |  | |  |  |  |
|  | **Median of computer and reading time** | | | **0** | **1** | **4** | **11** | |  | |  |  |  |
|  | **N(%)** |  | | **923(27.93)** | **866(26.20)** | **704(21.30)** | **812(24.57)** | |  | |  |  |  |
|  | Systolic blood pressure (mmHg) | Model 1 | | 128.5  (127.3, 129.7) | 127.2  (126.1, 128.2) | 126.5  (125.2, 127.7)* | 126.0  (124.8, 127.3) † | | 0.02 | |  |  |  |
|  |  | Model 2 | | 128.3  (127.1, 129.4) | 126.9  (125.8, 128.0) | 126.5  (125.2, 127.7)* | 126.6  (125.3, 127.8) | | 0.14 | |  |  |  |
|  |  | Model 3 | | 128.1  (127.0, 129.3) | 126.9  (125.8, 127.9) | 126.7  (125.5, 127.8) | 126.5  (125.3, 127.8) | | 0.14 | |  |  |  |
|  | Diastolic blood pressure(mmHg) | Model 1 | | 76.2 (75.5, 76.9) | 76.6 (76.0, 77.2) | 76.2 (75.5, 76.9) | 75.6 (74.9, 76.3) | | 0.07 | |  |  |  |
|  |  | Model 2 | | 76.4 (75.7, 77.1) | 76.7 (76.1, 77.3) | 76.1 (75.4, 76.8) | 75.4 (74.7, 76.1) | | 0.01 | |  |  |  |
|  |  | Model 3 | | 76.4 (75.7, 77.0) | 76.8 (76.2, 77.4) | 76.2 (75.5, 76.9) | 75.3 (74.6, 76.0)* | | 0.004 | |  |  |  |
|  | HDL-c (mmol/L) | Model 1 | | 1.39 (1.37, 1.41) | 1.40(1.38, 1.42) | 1.43 (1.40, 1.45)* | 1.41 (1.39, 1.43) | | 0.26 | |  |  |  |
|  |  | Model 2 | | 1.39 (1.37, 1.42) | 1.40 (1.38, 1.42) | 1.43 (1.40, 1.45) | 1.41 (1.38, 1.43) | | 0.45 | |  |  |  |
|  |  | Model 3 | | 1.39 (1.37, 1.42) | 1.40 (1.38, 1.42) | 1.42 (1.40, 1.44) | 1.41 (1.39, 1.43) | | 0.26 | |  |  |  |
|  | LDL-c (mmol/L) | Model 1 | | 3.30 (3.24, 3.36) | 3.27 (3.21, 3.32) | 3.21 (3.15, 3.27)* | 3.21 (3.15, 3.27) | | 0.17 | |  |  |  |
|  |  | Model 2 | | 3.30 (3.24, 3.36) | 3.26 (3.21, 3.31) | 3.21 (3.15, 3.27)* | 3.22 (3.16, 3.28) | | 0.34 | |  |  |  |
|  |  | Model 3 | | 3.29 (3.23, 3.35) | 3.26 (3.21, 3.31) | 3.22 (3.15, 3.28) | 3.22 (3.16, 3.28) | | 0.35 | |  |  |  |
|  | Cholesterol (mmol/L) | Model 1 | | 5.30 (5.23, 5.36) | 5.29 (5.23, 5.35) | 5.26 (5.19, 5.33) | 5.22 (5.15, 5.28) | | 0.23 | |  |  |  |
|  |  | Model 2 | | 5.29 (5.23, 5.36) | 5.28 (5.22, 5.34) | 5.26 (5.19, 5.33) | 5.23 (5.16, 5.30) | | 0.47 | |  |  |  |
|  |  | Model 3 | | 5.29 (5.22, 5.35) | 5.28 (5.22, 5.34) | 5.27 (5.20, 5.34) | 5.23 (5.16, 5.30) | | 0.60 | |  |  |  |
|  | Fasting plasma glucose (mmol/L) | Model 1 | | 4.80 (4.74, 4.85) | 4.81 (4.76, 4.86) | 4.72 (4.66, 4.78) | 4.78 (4.73, 4.84) | | 0.88 | |  |  |  |
|  |  | Model 2 | | 4.80 (4.75, 4.86) | 4.81 (4.76, 4.86) | 4.72 (4.66, 4.77)* | 4.78 (4.72, 4.84) | | 0.82 | |  |  |  |
|  |  | Model 3 | | 4.79 (4.74, 4.85) | 4.81 (4.76, 4.86) | 4.73 (4.67, 4.78) | 4.78 (4.73, 4.84) | | 0.99 | |  |  |  |
|  | Triglycerides | Model 1 | | 1.10 (1.06, 1.14) | 1.10 (1.06, 1.14) | 1.11 (1.07, 1.16) | 1.06 (1.02, 1.10) | | 0.17 | |  |  |  |
|  | (mmol/L) | Model 2 | | 1.09 (1.05, 1.13) | 1.09 (1.05, 1.13) | 1.12 (1.08, 1.16) | 1.08 (1.04, 1.12) | | 0.55 | |  |  |  |
|  |  | Model 3 | | 1.09 (1.05, 1.13) | 1.09 (1.05, 1.12) | 1.13 (1.09, 1.17) | 1.08 (1.04, 1.11) | | 0.60 | |  |  |  |
|  | hsCRP (mg/L) | Model 1 | | 1.11 (1.02, 1.21) | 1.04 (0.96, 1.12) | 1.03 (0.94, 1.12) | 1.04 (0.95, 1.13) | | 0.45 | |  |  |  |
|  |  | Model 2 | | 1.12 (1.02, 1.22) | 1.03 (0.95, 1.11) | 1.03 (0.94, 1.12) | 1.05 (0.96, 1.14) | | 0.62 | |  |  |  |
|  |  | Model 3 | | 1.10 (1.01, 1.18) | 1.02 (0.95, 1.09) | 1.06 (0.98, 1.15) | 1.05 (0.96, 1.14) | | 0.68 | |  |  |  |
|  | High-molecular weight adiponectin (µg/mL) | Model 1 | | 1.11 (1.05, 1.17) | 1.12 (1.06, 1.18) | 1.11 (1.05, 1.18) | 1.13 (1.06, 1.19) | | 0.37 | |  |  |  |
|  |  | Model 2 | | 1.10 (1.04, 1.17) | 1.13 (1.07, 1.19) | 1.11 (1.05, 1.18) | 1.12 (1.05, 1.18) | | 0.59 | |  |  |  |
|  |  | Model 3 | | 1.11 (1.05, 1.17) | 1.13 (1.08, 1.19) | 1.10 (1.04, 1.16) | 1.12 (1.06, 1.19) | | 0.52 | |  |  |  |
|  | Total adiponectin (µg/mL) | Model 1 | | 3.36 (3.24, 3.49) | 3.38 (3.26, 3.50) | 3.33 (3.21, 3.47) | 3.40 (3.27, 3.53) | | 0.31 | |  |  |  |
|  |  | Model 2 | | 3.35 (3.23, 3.48) | 3.39 (3.28, 3.51) | 3.34 (3.21, 3.47) | 3.39 (3.26, 3.52) | | 0.42 | |  |  |  |
|  |  | Model 3 | | 3.36 (3.24, 3.48) | 3.39 (3.28, 3.51) | 3.31 (3.19, 3.44) | 3.40 (3.27, 3.53) | | 0.33 | |  |  |  |
|  | HOMA-IR | Model 1 | | 1.29 (1.23, 1.35) | 1.23 (1.17, 1.28) | 1.27 (1.21, 1.34) | 1.27 (1.21, 1.33) | | 0.61 | |  |  |  |
|  |  | Model 2 | | 1.29 (1.23, 1.35) | 1.22 (1.17, 1.28) | 1.27 (1.21, 1.34) | 1.28 (1.22, 1.35) | | 0.37 | |  |  |  |
|  |  | Model 3 | | 1.28 (1.22, 1.33) | 1.22 (1.17, 1.27) | 1.30 (1.24, 1.35) | 1.27 (1.21, 1.33) | | 0.44 | |  |  |  |
| Model 1: Adjusted for age, sex, ethnicity, education | | | | | | | |  | |  | | | |
| Model 2: Model 1 further adjusted for TV viewing time, employment status, smoking, alcohol, parental history of diabetes, parental history of hypertension | | | | | | | | | |  | | | |
| Model 3: Model 2 further adjusted for potential mediators including total physical activity, BMI, ratio of polyunsaturated-to-saturated fat intake, and intake of total energy, fibre, cholesterol, carbohydrate and protein | | | | | | | | | |  | | | |
| HDL-c, fasting plasma glucose, triglycerides, hsCRP, high-molecular weight adiponectin, total adiponectin,and HOMA-IR were log transformed and the adjusted means were back transformed .  *p value ≤ 0.05, †p value ≤ 0.01  ¶p for trend: p value of linear regression for association of computer/reading time (as a continuous variable) and outcome variables. | | | | | | | | | |  | | | |
